# Surgical Repair of Skull Base CSF Leaks after Cisternography Diagnosis: Analysis of Validity and Surgical Outcome and Impact on Future Treatment Strategies

**DOI:** 10.1155/2022/8740352

**Published:** 2022-04-29

**Authors:** Christine Steiert, Luisa M. Kraus, Roland Roelz, Horst Urbach, Juergen Beck, Stephan Meckel, Juergen Grauvogel

**Affiliations:** ^1^Department of Neurosurgery, Medical Center-University of Freiburg, Faculty of Medicine, University of Freiburg, Freiburg 79106, Germany; ^2^Department of Neuroradiology, Medical Center-University of Freiburg, Faculty of Medicine, University of Freiburg, Freiburg 79106, Germany; ^3^Institute of Diagnostic and Interventional Neuroradiology, RKH Kliniken Ludwigsburg-Bietigheim, Ludwigsburg 71640, Germany

## Abstract

Skull base cerebrospinal fluid (CSF) leaks can lead to severe complications and require appropriate diagnosis and treatment. Cisternography is applied when exact localization via conventional imaging is not successful. The present study is aimed at identifying factors with potential impact on radiological results and surgical success. Cisternography followed by surgical repair due to skull base CSF leaks was performed in 63 cases between 2002 and 2020. The clinical and radiological findings were analyzed retrospectively. The etiology of CSF leaks was traumatic in 30.2%, spontaneous in 36.5%, and iatrogenic in 33.3%. The sensitivity of cisternography was 87.9%. Spontaneous CSF leaks tended to be diagnosed less frequently via cisternography and were significantly less frequently localized intraoperatively. The median postoperative follow-up period was 34 months. The primary surgical success rate was 79.4%, with a significantly higher success rate for lateral than for anterior skull base defects. Surgical failure tended to be lower in iatrogenic and higher in traumatic defects. Cisternography proved to be a highly sensitive method to localize skull base CSF leaks and can be recommended for advanced diagnostics. Iatrogenic leaks seemed to be more likely to have a favorable surgical outcome, whereas traumatic leaks tended to have a lower surgical success rate.

## 1. Introduction

Skull base cerebrospinal fluid (CSF) leaks represent an abnormal connection between the subarachnoid space and the sinonasal or tympanomastoid cavities. They occur as a result of a combined bony and dural defect and result in CSF rhinorrhea or otorrhea [1]. The most common complications are meningitis and pneumocephalus [2]. In untreated CSF leaks, meningitis can develop in 25–50% with a mortality of up to 10%, which is the reason as to why appropriate diagnosis and treatment are mandatory [3–5]. According to their etiology, CSF leaks are often categorized as traumatic (80%), iatrogenic (16%), or spontaneous leaks (3–4%) [6–8]. Spontaneous leaks may occur, for example, in association with tumors of the skull base, congenital deformities, or chronically increased intracranial pressure, especially in idiopathic intracranial hypertension [2].

For the primary diagnosis of CSF leaks, noninvasive high-resolution computed tomography (CT) and magnetic resonance (MR) imaging are used as complementary imaging modalities with a sensitivity of up to 89% [9–12]. However, this does not always allow definitive detection, such as in the presence of multiple bone defects or meningoceles, in the absence of bony defects, or in specific iatrogenic leaks [11, 13]. For these scenarios, CT or MR cisternography is required as an invasive diagnostic procedure [10, 11, 14]. While high-resolution CT cisternography is particularly suitable for visualizing associated bony defects, MR cisternography has advantages in detecting spontaneous leaks and associated encephaloceles [15–19]. The stated sensitivity of 80–95% is reported to be enhanced by combining these modalities [16, 17, 20].

Success rates of primary surgical repair of skull base CSF leaks vary from 80 to 90%, depending on the etiology, location, and extent of the leaks and the chosen surgical approach [21–23]. Within the last 20 years, endoscopic repair has become increasingly important due to reduced morbidity and high success rates [24–27]. Persistent or recurrent liquorrhea after previous surgical repair is defined as a secondary leak. These are reported to be more likely to appear with an iatrogenic or spontaneous etiology, with a success rate of 80–100% after reoperation [28].

The current study evaluates patients who underwent cisternography due to failure of a prior noninvasive diagnostic workup for the detection of CSF leaks, subsequently followed by surgical repair. In addition to the validity of cisternography in comparison with intraoperative findings, potential risk factors for the occurrence of secondary CSF leaks are investigated. Evaluation of the congruence of cisternography with intraoperative results is of particular interest. High congruence and, thus, precise preoperative defect localization would allow increased use of minimally invasive surgical approaches with reduced morbidity in the future, and risk analysis may reveal factors that tend to argue against such therapeutic approaches.

## 2. Materials and Methods

### 2.1. Patient Characteristics and Study Design

The study included all consecutive patients who underwent cisternography for a suspected skull base defect followed by surgery between 01/2002 and 06/2020. The indication for cisternography was given if there was a clinically clear and/or laboratory-chemically proven (positive result for beta-2 transferrin) liquorrhea, and conventional imaging did not provide clear evidence of the underlying defect. In the presence of multiple bony defects or iatrogenic liquorrhea, cisternography was used to precisely localize the underlying defect in order to minimize the extent of (revision) surgery, e.g., in case of mastoid fluid collection after acoustic neuroma surgery to differentiate between intra- and extradural leakage. Persistent or recurrent liquorrhea after surgical repair was also an indication, including when previous surgery had been performed without prior cisternography because of sufficient evidence of the defect on conventional imaging, such as in trauma with skull base fractures or in a spontaneous etiology in the presence of a meningocele.

The retrospective evaluation was based on medical records as well as surgical and radiological reports. The primary endpoint of this study was to determine the sensitivity and specificity of cisternography when compared with intraoperative findings, including consideration of, for example, the etiology and localization of the defect and the different conditions of the examination. Secondary endpoints were possible factors influencing surgical success, e.g., the etiology and localization of the defect, the surgical approach, perioperative management, or patient-specific factors. Every surgery was performed in the Department of Neurosurgery as a tertiary referral center. Informed consent for the radiological examination and the surgical procedure was obtained from all patients or their legal representative. The retrospective analysis was approved by the independent ethics committee of our medical center (reference no. 21-1248_1) and is reported according to institutional guidelines.

### 2.2. Cisternography Procedure

The standard procedure was CT cisternography, and the decision regarding whether to perform additional MR cisternography (if available) was made individually. After cervical or lumbar application of the iodine-containing contrast agent under seizure prophylaxis (and additional application of gadolinium, if MR cisternography was planned), patients were prone-positioned (30–40° trendelenburg) and underwent CT scans 10–20 minutes thereafter. In the case of additional MR cisternography, MR images were performed in the supine position, usually 1.5–3.5 hours after contrast agent application.

### 2.3. Surgical Procedure

All surgical procedures were performed under general anesthesia. The choice of surgical approach was dependent on the localization and extent of the defect as well as on the previous history (especially in iatrogenic defects), with minimally invasive therapeutic approaches preferred whenever possible. Defects at the frontal sinus, the petrous bone or the mastoid required (re)craniotomy, whereas surgical repair of sellar or clival defects was performed exclusively via endoscopic endonasal approaches. When localized at the cribriform plate, the planum sphenoidale, or the anterior temporo-basal region (adjacent to the sphenoid sinus) an endonasal approach was usually chosen for smaller, centrally located defects, whereas a transcranial approach was typically selected for more extensive defects with lateral extension. In patients with evident liquorrhea but negative cisternography, the surgical procedure had to be planned as best as possible based on the findings of conventional imaging, clinical presentation, and previous history (with some uncertainty remaining for the patient, albeit unavoidable due to liquorrhea).

### 2.4. Statistical Analysis

Methods of descriptive statistics were used. Categorical data are presented as absolute and relative frequencies (in %). For numerical data, median values with the minimum/maximum and the interquartile range (IQR) were calculated (as appropriate). The validity of the cisternography was determined via binary classification (calculation of sensitivity and specificity). The influence of risk factors was examined via the chi-square test of independence. The significance level was set to *p* < 0.05. Statistical analysis was performed using GraphPad Prism software version 9.1.1 for Mac (GraphPad Software, San Diego, CA, USA).

## 3. Results

### 3.1. Patient Characteristics

A total of 63 cisternography examinations followed by surgery were performed in 53 patients (27 female, 26 male, median age 45.0 years (IQR 34–57, range 5–76 years)). Forty-four patients underwent this procedure once. Due to persistent liquorrhea, eight patients underwent it twice and one patient three times. CSF leaks presented with rhinoliquorrhea in 79.3%, with otoliquorrhea in 19.1%, and with both in 1.6%. Prior beta-2 transferrin testing was performed in 61.9% (79.5% positive results). Prior meningitis or cerebral abscess occurred in 25.4%, significantly more often in anterior temporo-basal defects (31.2% versus (vs.) 6.4%, *p* = 0.01). The etiology of CSF leaks was traumatic in 30.2% (19 procedures in 14 patients), spontaneous in 36.5% (23 procedures in 20 patients), and iatrogenic in 33.3% (21 procedures in 19 patients). Nineteen patients had undergone previous CSF leak repair without prior cisternography, seven for traumatic liquorrhea and six each for spontaneous or iatrogenic liquorrhea. In these, cisternography and subsequent surgery were performed because of secondary CSF leaks. Detailed information on the patient characteristics, categorized by the etiology of liquorrhea, is listed in Table 1.

### 3.2. Cisternography Results and Intraoperative Findings

**Figure 1:**
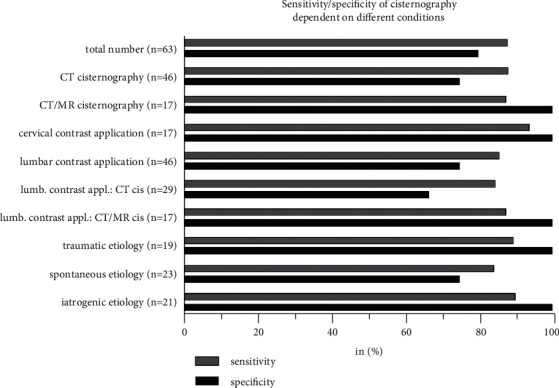
Sensitivity and specificity of cisternography dependent on modality, site of contrast application, and etiology of liquorrhea. *n*: number; CT cisternography (cis): computed tomography cisternography; CT/MR cisternography (cis): combined computed tomography and magnetic resonance cisternography; lumb. contrast appl.: lumbar contrast application.

**Figure 2:**
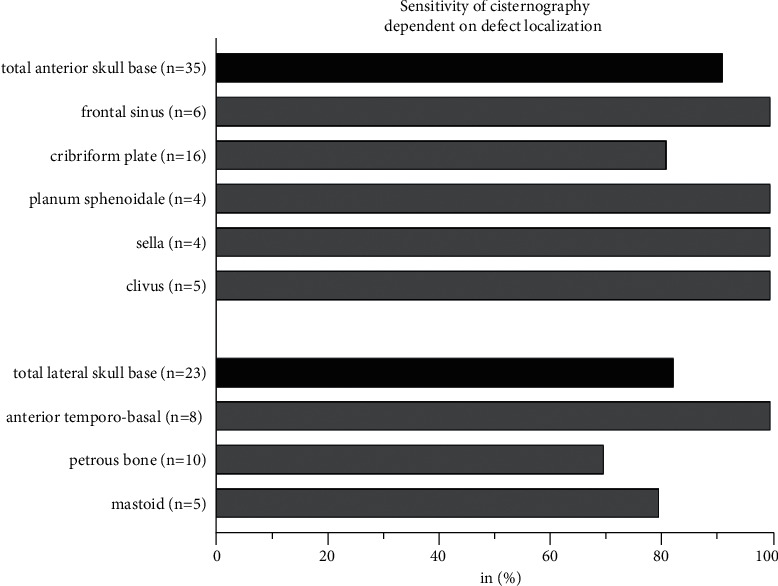
Sensitivity of cisternography dependent on defect localization. All cases positive for intraoperative defect (*n* = 58) with or without cisternography detection; dark-gray bars: sensitivity of total numbers of anterior or lateral skull base defects; light-gray bars: sensitivity of distinct defect localizations.

The median time between cisternography and surgery was three days (IQR 2.0–7.5, range 0–230). For the total of 63 cisternography examinations, there was a sensitivity of 87.9% and a specificity of 80.0%, comparing radiological with intraoperative findings. The contrast agent was applied via a lumbar puncture (or drainage) in 73.0% and a cervical puncture in 27.0%. The sensitivity of cisternography for cervical vs. lumbar contrast application was higher at 93.8% vs. 85.7%, respectively, albeit without a significant difference. Combined CT/MR cisternography was performed in 27.0%. There was neither a significant difference between the sensitivity of CT vs. combined CT/MR cisternography, nor of the radiological results depending on the etiology. Detailed information on sensitivity/specificity is given in Figures 1 and 2.

A spontaneous etiology tended to have lower cisternography detection rates (73.9% vs. 87.5%), and significantly fewer defects were diagnosed intraoperatively in spontaneous than in traumatic or iatrogenic CSF leaks (82.6% vs. 97.5%, *p* = 0.035). In 82.5%, the detected defects could be confirmed by the intraoperative findings. In 11.1%, no defect was detected via cisternography but was found intraoperatively. In 6.4%, a negative cisternography correlated with negative intraoperative findings. In secondary CSF leaks, the prevalence of frontal sinus defects was significantly higher in comparison to other locations (66.7% vs. 26.3%, *p* = 0.041). Detailed information on the radiological and clinical results, categorized by the etiology of liquorrhea, is listed in Tables 1 and 2.

### 3.3. Surgery and Surgical Outcome

**Figure 3:**
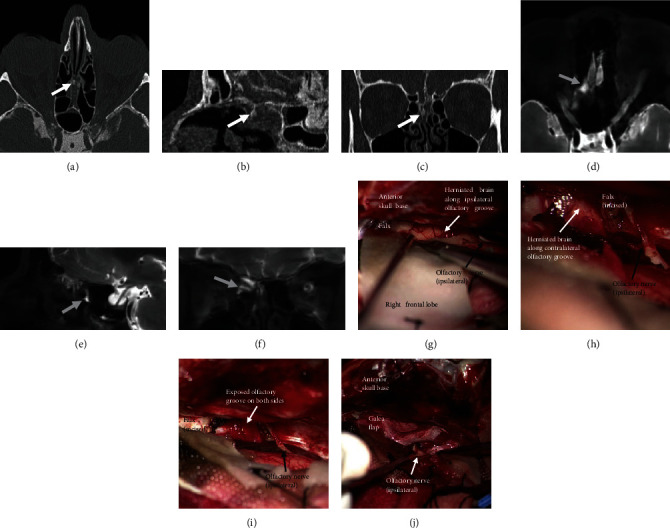
Case 1: 61-year-old female patient with spontaneous liquorrhea due to a CSF leak at the cribriform plate. CT cisternography in axial (a), sagittal (b), and coronar (c) views demonstrating contrast leakage at the cribriform plate (white arrows). MR cisternography in axial (d), sagittal (e), and coronar (f) views demonstrating the delayed accumulation of the contrast agent in the ethmoid (light-gray arrows). (g–j) Intraoperative microscopic view showing the anterior skull base via a transcranial right frontolateral approach; (g) brain tissue is deeply herniated along the defect at the ipsilateral olfactory groove; (h) after ipsilateral elevation of the herniated brain from the olfactory groove and partial resection of the falx also the contralateral herniation is visible; (i) the olfactory groove is now exposed on both sides; (j) the olfactory groove is covered on both sides in a multilayer fashion while preserving the olfactory nerves.

**Figure 4:**
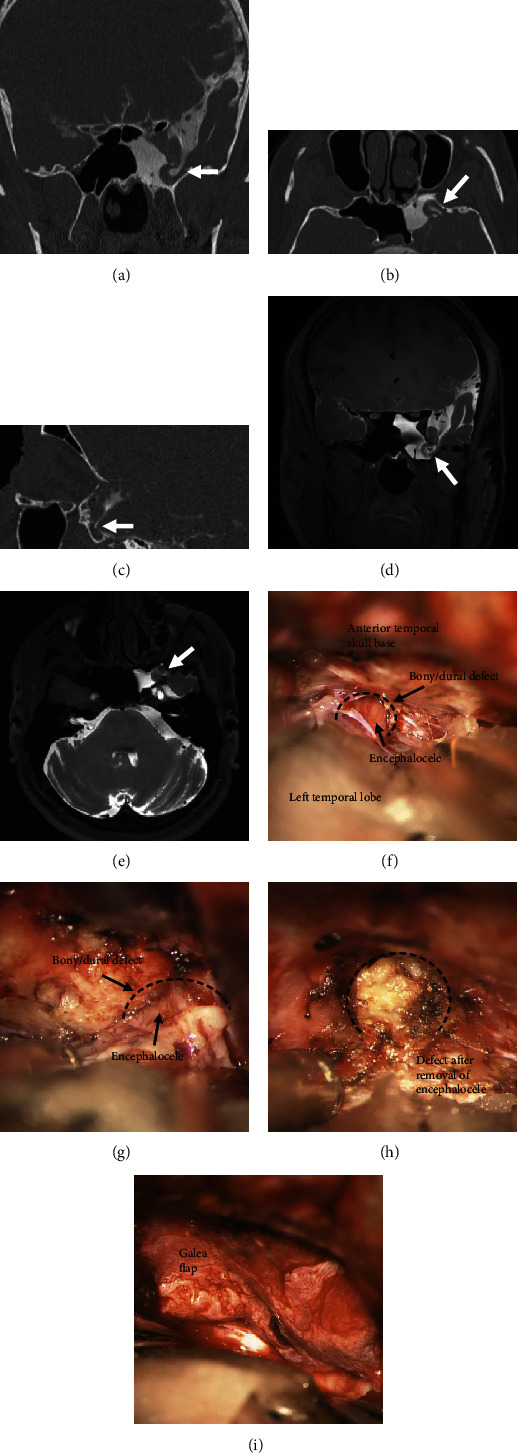
Case 2: 45-year-old male patient with spontaneous liquorrhea due to an anterior temporo-basal CSF leak with an associated encephalocele in the sphenoid. CT cisternography in coronar (a), axial (b), and sagittal (c) views demonstrating the skull base defect with contrast leakage along the encephalocele and the accumulation of the contrast agent in the sphenoid (white arrows). MR cisternography in coronar (d) and axial (e) views allowing precise visualization of the encephalocele and the surrounding brain tissue (white arrows). (f–i) Intraoperative microscopic view showing the anterior temporal skull base via a transcranial left pterional approach; (f, g) Exposure of the defect (dotted black line) at the anterior temporal skull base with the associated encephalocele; (h) defect (dotted black line) after removal of the encephalocele; (i) the defect area is covered in a multilayer fashion.

Depending on the defect localization and extent, as well as on the previous history, a transcranial approach was chosen in 66.7% and an endonasal approach in 33.3% (see Table 2). Different combinations of autologous tissue and alloplastic materials were used for multi-layer repair. Preoperative application of fluorescein via lumbar drainage so as to improve intraoperative defect localization was performed in 12.7% (87.5% endonasal, 12.5% transcranial). For an average of five days, 71.4% of cases underwent postoperative CSF draining (65.1% via a lumbar and 6.3% via a ventricular drainage). Figures 3 and 4 show examples of the radiological findings and the corresponding intraoperative results.

The median follow-up period was 34 months (IQR 6–96, range 0–179 months). Liquorrhea persisted or recurred postoperatively in 12 patients. One patient refused further interventions and was lost to follow-up, liquorrhea was resolved with lumbar drainage in two patients, and successful leak repair was achieved via another surgery following a repeated cisternography in nine patients (one patient twice).

Different potential risk factors for postoperative persistence or recurrence of liquorrhea were investigated. There was a significantly lower rate of persistent/recurrent liquorrhea when cisternography had revealed a lateral skull base defect (5.3% vs. 27.3%, *p* = 0.048). A tendency for an increased risk of persistent/recurrent liquorrhea was observed in the group without cisternography defect detection (36.4% vs. 17.3%) and in those with defect localization at the cribriform plate (35.7% vs. 16.3%), but without statistical significance. Furthermore, the group with a traumatic etiology was more likely to have persistent or recurrent liquorrhea (31.6% vs. 15.9%), as was the group with a body mass index (BMI) between 25 and 30 (33.3% vs. 14.3%).

Definitive cisternography defect detection tended to increase the chance of a permanent absence of liquorrhea after surgery (82.7% vs. 63.6%), as did an iatrogenic etiology (90.5% vs. 73.8%), a defect localization at the petrous bone (100.0% vs. 76.8%), and a short time interval of less than two days between cisternography and surgery (92.9% vs. 75.5%). Detailed information is listed in Table 3.

### 3.4. Complications

After cisternography, one case of a self-limiting seizure (1.6%) and three cases of severe headaches lasting up to three days (4.8%) were observed. After transcranial surgery, one case (1.6%) each of meningitis, temporary aphasia due to edema in the adjacent temporal lobe, autolysis of the bone flap, and wound infection occurred. After endonasal surgery, there was one case (1.6%) each of meningitis, new hyposmia, and a mucocele.

## 4. Discussion

Skull base CSF leaks can be caused by several mechanisms. About 80% are the result of craniofacial trauma with skull base fractures, where the leaks are mainly located at the frontal sinus, sphenoid sinus, or cribriform plate [7, 8, 29]. Approximately 16% of CSF leaks are iatrogenic and related to endoscopic sinus surgery or neurosurgical operations [30]. The remaining CSF leaks occur spontaneously, e.g., in the context of skull base abnormalities, bony erosion, or increased intracranial pressure such as idiopathic intracranial hypertension [31]. Due to the high risk of developing meningitis or other complications, surgical intervention is usually mandatory, and precise preoperative localization is reported to be associated with a significantly higher surgical success rate and may also facilitate the use of minimally invasive therapeutic approaches [22, 32]. When noninvasive CT or MR imaging does not allow precise localization of a CSF leak, cisternography is used for further diagnostic evaluation [10, 11]. The long-established method of CT cisternography has certain limitations, for example, due to the similar appearance of contrast-enriched CSF and bone, or in cases of spontaneous leakage with associated cephaloceles. Therefore, MR cisternography has been established as a method offering diagnostic advantages in such scenarios, with high-resolution isotropic datasets providing a high contrast between bone structures, air cells, and gadolinium-enriched CSF [15, 18, 33]. Various studies describe an extremely low risk profile of intrathecal gadolinium application, which continues to be off-label use [19, 34, 35]. Longer follow-up data after intrathecal gadolinium application are now also available, supporting the safety of the technique [19, 36].

Because of their invasiveness, CT and MR cisternography are reserved for patients with failure of a prior noninvasive diagnostic workup [10, 37]. The sensitivity of the individual methods or of their combination is generally reported to be >80%; however, there is a lack of information on how cisternography results are verified and whether sensitivity differs depending on the specific study conditions or the patient groups [14, 17, 20]. In our study, the radiological result was always cross-checked with the subsequent intraoperative findings. Furthermore, the sensitivity of cisternography was compared in the context of different study conditions and patient groups. All of our selected patients met the criterion of a clinically and/or laboratory-chemically proven CSF leak and “failure of a prior noninvasive diagnostic workup,” also including patients with secondary CSF leaks.

Our groups of traumatic, spontaneous, or iatrogenic leaks were approximately equal in size. Specific patient characteristics regarding gender and BMI depending on the etiology of liquorrhea (see Table 1) were consistent with descriptions of patients most commonly affected by traumatic brain injury or by spontaneous CSF leaks [21, 30, 38, 39]. Corresponding to the regions frequently affected in trauma, cisternography revealed significantly more cases with anterior than lateral skull base defects in the traumatic group. In contrast, significantly more cases with lateral defects were found in the iatrogenic group (by including complex postoperative leaks, e.g., after tumor resection in the cerebellopontine angle), which explains the significantly high rate of otoliquorrhea and the low rate of rhinoliquorrhea in comparison with the other groups. The significantly high prevalence of frontal sinus defects in cases with prior CSF leak repair before cisternography also reflects the inclusion of patients with secondary leaks resulting from complex traumatic defects. Interestingly, spontaneous leaks could be detected intraoperatively significantly less often than traumatic or iatrogenic leaks, and spontaneous leaks tended to have lower cisternography detection rates, associated with a lower sensitivity of cisternography. Several authors have described the subgroup of spontaneous leaks, harboring defects particularly at the sphenoid and ethmoid or laterally at the temporal skull base, where special requirements must be addressed such as the associated increased intracranial pressure [30, 39, 40]. The recurrence rate after surgical repair of spontaneous leaks was reported to be higher in comparison with other etiologies [38–40]. However, the recurrence rate could be partially reduced by advances in endoscopic techniques and by knowledge and treatment of related risk factors [28, 39]. An association with an increased recurrence rate in our group with spontaneous leaks was not found, but a tendency toward an increased rate of recurrence in patients without cisternography defect detection (the spontaneous etiology tended to have lower cisternography detection rates than did other etiologies, and showed significantly lower intraoperative detection rates).

The overall sensitivity of cisternography of 87.9% in our study population is similar to that reported by other authors [10, 14, 20]. There were no significant differences in sensitivity depending on the etiological subgroups or on the modality used. Sensitivity with cervical contrast application tended to be higher than with lumbar application. This could possibly be attributed to the fact that the contrast agent is applied closer to the target site in a cervical puncture, but the group size was relatively small, and there was no significant difference. The higher risks of a cervical puncture should also be considered, although the complication rate in our population was extremely low, and no specific problems dependent on the site of the puncture occurred after cisternography. The sensitivity of cisternography tended to be higher in anterior than in lateral skull base defects. In particular, lateral defects after previous intradural surgery were associated with lower sensitivity, possibly related to arachnoid adhesions due to scarring and, thus, suboptimal subarachnoid contrast distribution. Cisternography has been performed at a high level in our neuroradiology department during the last two decades, as reflected in the high quality of the examination results. Continuous work is being done on further development, e.g., on refining the MR cisternography technique, with improvements in sensitivity, e.g., through the acquisition of high-resolution isotropic datasets [19, 36, 37, 41]. A recent publication on preoperative imaging techniques prior to endonasal endoscopic repair of skull base leaks also reports high sensitivity with the combination of CT cisternography and magnetic resonance hydrography [32].

Our number of 63 cases with skull base CSF leaks diagnosed via cisternography and subsequently undergoing surgery is rather high. Furthermore, the subdivision of our cases into equally distributed etiological groups is a specific feature in comparison with the literature. In often smaller series, other authors rather report only the results of a single etiological subgroup or a single surgical approach, and leak detection is commonly attempted by conventional imaging (and not via cisternography) [14, 22, 40, 42, 43]. Moreover, our median follow-up period of 34 months is quite long, which should also be considered regarding the success rates.

There are numerous reports on the methodology and the success rates of surgical repair of skull base CSF leaks [14, 25, 40]. The increasing development of endoscopic repair techniques has had a major impact by, if possible (depending on the location and type of the defect), avoiding more invasive transcranial surgery. Therefore, interdisciplinary collaboration has become more important, particularly between neurosurgeons and otolaryngologists [44]. Primary success rates vary from 60 to 100% in older publications, with an average of 90% in a review of 14 papers (published between 1990 and 1999) by Hegazy et al. [26]. A more recent review by Psaltis et al., dealing with 55 papers on endoscopic repair of CSF leaks, describes an overall primary surgical success rate of 90% and a lower complication rate in comparison with transcranial procedures [24, 27]. Similar success rates are given in various original works [30, 38, 43, 45]. In this context, the importance of precise localization of defects should be emphasized.

In our selected group of patients, the primary surgical success rate was 79.4%, and after repeated cisternography and surgery, the rate was 100%. Our specific population renders comparisons with other authors difficult. Konuthula et al. published a review dealing with secondary CSF leaks (those that recurred after an initial endoscopic repair) and described rates of primary success being around 80% and those of success after reoperation being from 80 to 100% [28]. The numbers are similar to ours, although the inclusion criterion in this review was the previous endoscopic procedure with respective limitations regarding the localization of the defect.

The influence of postoperative CSF drainage is controversially discussed in the literature. Retrospective and randomized studies provide evidence of both a positive effect and no effect on the surgical outcome [40, 46–48]. In our retrospective series, no significant effect of postoperative CSF drainage was observed. What is more, the surgical outcome was found to be independent of the chosen approach and the material used for coverage. Detailed information is listed in Table 3.

The rate of persistent or recurrent liquorrhea after surgery was significantly lower when cisternography revealed a lateral (compared with an anterior) skull base defect. The group with lateral skull base defects included many patients with iatrogenic leaks. This probably presented more favorable conditions for surgical repair, since these were usually less complex defects in a primarily limited area, even though the sensitivity of cisternography tended to be lower here (as discussed above). In contrast, the group with anterior skull base defects included many traumatic defects, which tended to be more complex, possibly influencing the rather lower surgical success rate.

The total number of cases of postoperative persistent or recurrent liquorrhea was low (*n* = 13). This probably contributes to a certain bias in statistical testing, which showed some tendencies regarding potential factors influencing the surgical success rate, albeit mostly without statistical significance. For example, there appeared to be a positive effect on the surgical success rate in patients with definitive defect localization via cisternography, whereas a negative examination tended to show an unfavorable effect. There was also a tendency for a higher success rate when surgery was performed on the day after cisternography, rather than subsequently. This may be related to the formation of arachnoid adhesions within days or weeks after cisternography, masking the overall defect extent intraoperatively. The potentially lower success rate for defects at the cribriform plate and the higher success rate for defects at the petrous bone may be attributed to the fact that the former were more likely to be found in complex trauma cases and the latter in more favorable iatrogenic cases (as discussed above).

The retrospective study design with the inclusion of cases over the last 18 years is certainly a limitation. Thus, depending on the individual expertise, several neuroradiologists assessed the cisternography examinations, as well as several neurosurgeons deciding on and performing the surgical intervention. We chose not to retrospectively reassess the cisternography results, because surgical management decisions were based on the findings produced at that time; therefore, the validity of the current evaluation depends on them. The different etiologies and types of CSF leaks within our heterogeneous patient group cause some difficulty, although interesting aspects are also revealed by comparing the different etiological groups. The total number of cases with postoperative persistent or recurrent liquorrhea is relatively small. Therefore, the statistical power regarding the potential factors influencing the surgical success rates is certainly limited.

## 5. Conclusions

Cisternography proved to be a highly sensitive method to localize skull base CSF leaks of different etiologies and, therefore, can be generally recommended for advanced diagnostics, although spontaneous leaks still seem to be rather underdiagnosed. The incidence of successful primary surgical repair was significantly higher in lateral than in anterior skull base defects, probably associated with a more favorable outcome of iatrogenic versus traumatic leaks. Based on our results, cisternography presents as an excellent diagnostic method for precisely and reliably localizing even complex CSF leaks, thus enabling increased use of focused minimally invasive surgical approaches as a future perspective.

## Figures and Tables

**Table 1:** Patient characteristics categorized by etiology of CSF leak.

Etiology of liquorrhea		Number of procedures (cisternography and surgery due to liquorrhea) (%, level of significance)		
		Traumatic: 19 (30.2)	Spontaneous: 23 (36.5)	Iatrogenic: 21 (33.3)
Sex	Female	6 (31.6, *p* = 0.045^∗^)	16 (69.6, *p* = 0.024^∗^)	10 (47.6, *p* = 0.722)
	Male	13 (68.4, *p* = 0.045^∗^)	7 (30.4, *p* = 0.024^∗^)	11 (52.4, *p* = 0.722)
BMI	<25	11 (57.9, *p* = 0.033^∗^)	3 (13.1, *p* = 0.002^∗^)	10 (47.6, *p* = 0.271)
	25-30	6 (31.6, *p* = 0.846)	9 (39.1, *p* = 0.459)	6 (28.6, *p* = 0.571)
	>30	2 (10.5, *p* = 0.037^∗^)	11 (47.8, *p* = 0.010^∗^)	5 (23.8, *p* = 0.554)
Presence of rhinoliquorrhea		17 (89.5, *p* = 0.258)	21 (91.3, *p* = 0.113)	13 (61.9, *p* = 0.006^∗^)
Presence of otoliquorrhea		2 (10.5, *p* = 0.193)	2 (8.7, *p* = 0.076)	9 (42.9, *p* = 0.002^∗^)
Presence of meningitis/brain abscess		4 (21.1, *p* = 0.603)	6 (26.1, *p* = 0.924)	6 (28.6, *p* = 0.682)
Cisternography defect detection	total^1^	17 (89.5, *p* = 0.341)	17 (73.9, *p* = 0.171)	18 (85.7, *p* = 0.639)
	Anterior skull base^2^	15 (78.9, *p* = 0.006^∗^)	12 (52.2, *p* = 0.980)	6 (28.6, *p* = 0.007^∗^)
	Lateral skull base^3^	2 (10.5, *p* = 0.026^∗^)	5 (21.7, *p* = 0.270)	12 (57.1, *p* < 0.001^∗^)
Intraoperative defect detection		19 (100.0, *p* = 0.126)	19 (82.6, *p* = 0.035^∗^)	20 (95.2, *p* = 0.510)
Intraoperative encephalo-/meningocele		4 (21.1, *p* = 0.313)	5 (21.7, *p* = 0.200)	0 (0.0, *p* = 0.022^∗^)

^1^Total number of skull base defects detected by cisternography (*n* = 52); ^2^defects detected at anterior skull base (*n* = 33); ^3^defects detected at lateral skull base (*n* = 19); ^∗^statistically significant difference.

**Table 2:** Cisternography defect localization and surgical approach categorized by etiology of CSF leak.

		Number of procedures (cisternography and surgery due to liquorrhea) (%)							
Etiology of liquorrhea		Traumatic: 19 (30.2)		Spontaneous: 23 (36.5)		Iatrogenic: 21 (33.3)		Total: 63 (100.0)	
Surgical approach		Transcranial: 12 (63.2)	Endonasal: 7 (36.8)	Transcranial: 14 (60.9)	Endonasal: 9 (39.1)	Transcranial: 16 (76.2)	Endonasal: 5 (23.8)	transcranial^1^: 42 (66.7)	endonasal^2^: 21 (33.3)
Cisternography defect localization	Frontal sinus	4 (21.1)	—	—	—	2 (9.5)	—	6 (9.5)	—
	Cribriform plate	3 (15.8)	2 (10.5)	6 (26.1)	2 (8.7)	—	1 (4.8)	9 (14.3)	5 (7.9)
	Planum sphenoidale	1 (5.3)	2 (10.5)	—	1 (4.3)	—	—	1 (1.6)	3 (4.8)
	Sella	—	—	—	1 (4.3)	—	3 (14.3)	—	4 (6.3)
	Clivus	—	3 (15.8)	—	2 (8.7)	—	—	—	5 (7.9)
	Anterior temporo-basal	—	—	4 (17.4)	1 (4.3)	2 (9.5)	1 (4.8)	6 (9.5)	2 (3.2)
	Petrous bone	2 (10.5)	—	—	—	5 (23.8)	—	7 (11.1)	—
	Mastoid	—	—	—	—	4 (19.0)	—	4 (6.3)	—
	w/o defect	2 (10.5)	—	4 (17.4)	2 (8.7)	3 (14.3)	—	9 (14.3)	2 (3.2)

w/o: without; ^1^78.6% performed by neurosurgeons alone and 21.4% performed interdisciplinarily by neurosurgeons and otorhinolaryngologists; ^2^57.1% performed by neurosurgeons alone and 42.9% performed interdisciplinarily by neurosurgeons and otorhinolaryngologists.

**Table 3:** Statistical analysis of potential factors influencing the risk of persistent or recurrent liquorrhea after surgery.

		Total number of procedures (%)	Number of persistent or recurrent CSF leak after surgery (%, level of significance)
Sex	Female	32 (50.8)	8 (25.0, *p* = 0.384)
	Male	31 (49.2)	5 (16.1, *p* = 0.384)
BMI	<25	24 (38.1)	5 (20.8, *p* = 0.976)
	25-30	21 (33.3)	7 (33.3, **p** = 0.078)
	>30	18 (28.6)	1 (5.6, **p** = 0.061)
Etiology	Traumatic	19 (30.2)	6 (31.6, **p** = 0.158)
	Spontaneous	23 (36.5)	5 (21.7, *p* = 0.870)
	Iatrogenic	21 (33.3)	2 (9.5, **p** = 0.123)
Meningitis/brain abscess (preoperative)		16 (25.4)	2 (12.5, *p* = 0.352)
Cisternography defect detection		52 (82.5)	9 (17.3, **p** = 0.156)
Anterior skull base		33 (52.4)	8 (24.2, *p* = 0.458)
Frontal sinus		6 (9.5)	1 (16.7, *p* = 0.801)
Cribriform plate		14 (22.2)	5 (35.7, **p** = 0.114)
Planum sphenoidale		4 (6.3)	1 (25.0, *p* = 0.824)
Sella		4 (6.3)	0 (0.0, *p* = 0.292)
Clivus		5 (7.9)	1 (20.0, *p* = 0.971)
Lateral skull base		19 (30.1)	1 (5.3, **p** = 0.048^∗^)
Anterior temporo-basal		8 (12.7)	1 (12.5, *p* = 0.543)
Petrous bone		7 (11.1)	0 (0.0, **p** = 0.152)
Mastoid		4 (6.3)	0 (0.0, *p* = 0.292)
Negative cisternography		11 (17.5)	4 (36.4, **p** = 0.156)
Preoperative application of fluorescein		8 (12.7)	2 (25.0, *p* = 0.744)
<2 days between cisternography and surgery		14 (22.2)	1 (7.1, **p** = 0.157)
Surgical approach	Transcranial	42 (66.7)	9 (21.4, *p* = 0.826)
	Endonasal	21 (33.3)	4 (19.0, *p* = 0.826)
Intraoperative defect detection		58 (92.1)	11 (19.0, *p* = 0.265)
Intraoperative encephalo-/meningocele		9 (14.3)	3 (33.3, *p* = 0.309)
Cisternography result = intraoperative result		55 (87.3)	11 (20.0, *p* = 0.744)
Materials used for multilayer repair	Fat	23 (36.5)	5 (21.7, *p* = 0.870)
	Muscle tissue	11 (17.5)	2 (18.2, *p* = 0.825)
	Galea	29 (46.0)	6 (20.7, *p* = 0.992)
	Fascia/mucosa	15 (23.8)	3 (20.0, *p* = 0.945)
	Alloplastic dural grafts	31 (49.2)	9 (29.0, *p* = 0.054)
	Bone graft or substitutes/titanium mesh	16 (25.4)	2 (12.5, *p* = 0.352)
Postoperative CSF draining		45 (71.4)	10 (22.2, *p* = 0.623)

^∗^Statistically significant difference; bold font: values with tendency toward statistical dependency without significance.

## Data Availability

The data used to support the findings of this study are available from the corresponding author upon request.
